# The efficacy and safety of intralesional *Candida* vaccine versus topical diphencyproprobenone in immunotherapy of verruca vulgaris: A randomized comparative study

**DOI:** 10.1007/s00403-022-02402-7

**Published:** 2022-10-17

**Authors:** Mohamed H. M. EL-Komy, Shahenda Gamal Shamma, Nermeen Ibrahim Bedair

**Affiliations:** 1 Department of Dermatology, Faculty of Medicine, Cairo University, Giza, Giza Egypt; 2 Department of Dermatology, Elmenshawy General Hospital, Tanta, Egypt; 3 Department of Dermatology, Andrology, Sexual Medicine and STDs, Faculty of Medicine, Helwan University, Cairo, Egypt; 4 Department of Dermatology and Andrology, Armed Forces College of Medicine, Sexual Medicine and STDs, Faculty of Medicine, Armed forces college of medicine, Cairo, Cairo Egypt

**Keywords:** Warts, HPV, Immunotherapy, Candida antigen, DPCP

## Abstract

Various therapeutic options are available for verruca. While physical destruction may be associated with scarring, immunotherapy may be effective in treating warts through stimulating body immune response. The objective of the study was to compare the efficacy, safety, and outcome of *Candida* antigen vs diphencyprone (DPCP) in the treatment of warts. Fifty patients were randomly assigned to receive either intralesional *Candida* antigen every 3 weeks or weekly DPCP application. Both treatments were applied only to the mother wart. Lesions’ clearance and associated side effects were observed up to 4 weeks after treatment. Two blinded physicians evaluated photos of warts before and 4 weeks after the end of treatment. Both modalities granted wart clearance and/or improvement with no statistically significant difference; however, *Candida* antigen was significantly better in clearing adjacent untreated warts (*p* = 0.046). Fewer side effects were observed among the *Candida* antigen group. The response was duration associated in the *Candida* groups only. Intralesional *Candida* antigen injection and DPCP treatments for warts yielded improvement with superiority of *Candida* injection in eradicating distant lesions and fewer side effects. A shorter wart duration may be associated with a better therapeutic response with *Candida* antigen.

**Supplementary Information:**

The online version contains supplementary material available at 10.1007/s00403-022-02402-7.

## Background

Human papilloma virus (HPV)-induced verruca are benign proliferation that may occur at any part of the skin. [1] Verruca treatment includes topical keratolytics, cryotherapy, electrocautery, chemical cautery, and laser ablation. [2] Such modalities can be painful and time consuming, and none of them is considered as gold standard due to potential scarring, disfigurement, and recurrence.

The mechanism of action of immunotherapy in wart treatment is not yet clear; however, it may boost the immune system to recognize the antigen via the delayed hypersensitivity reaction and subsequently clear the HPV [3]. Hence, it also has a potential to resolve distant warts, possibly by proliferation of HPV-specific mononuclear cells [5].

Candida was the first injectable antigen to be reported as a successful option in the treatment of warts [6]. Topical immunotherapy with diphenylcyclopropenone (DPCP) has also showed efficacy in topically treating recalcitrant warts [7]. In the present study, we compared two different modalities of immunotherapy, namely intralesional *Candida* antigen versus contact DPCP in terms of efficacy and safety for the treatment of verruca.

## Materials and methods

This study was conducted between July 2019 and June 2020 at the outpatient clinic of the dermatology department of Badr University Hospital, Helwan University. The study protocol was approved by the Faculty of Medicine, Helwan University Research Ethics Committee. All the participants received full information prior to enrollment and an informed consent was obtained. Fifty patients were recruited, either with recalcitrant or non-recalcitrant warts. Warts were considered recalcitrant, when they failed to clear following treatment with two or more different modalities. We excluded patients receiving any immune-altering drugs (e.g., systemic steroids, chemotherapy) as well as those with a history of any disorder affecting their immune system, e.g., HIV and diabetes, pregnant and lactating females, patients with atopic dermatitis or history of other allergic disorders, and patients with history of wart treatment within 12 weeks prior to enrollment were also excluded.

During the first visit following recruitment, full history was obtained, thorough clinical examination was performed, and warts’ lesions were photographed with detailed data documentation. Participants were randomly assigned to group A or B using the sealed envelope technique.

### Group A (candida antigen group)

Before inclusion into this group, patients were injected with 0.1 ml (ml) of 1/1000 purified *Candida* antigen solution (manufacturer: Ain Shams University Hospital, Specific Desensitizing Vaccine unit; manufacture date: March 2018, expiry date: March 2021) intradermally into the flexor aspect of the forearm using 1 ml insulin syringes. After 48–72 h, the existence of a visible cutaneous reaction in the form of erythema and induration of at least 5 mm in diameter was considered as positive sensitization response to the *Candida* antigen. All the enrolled patients showed a positive response to the *Candida* antigen and proceeded to the next step of starting active therapy. All recruits in this group were then injected with 0.1 ml of the solution in only the mother wart (the earliest or initial wart on the body). If the patient was not sure about the initial wart, then the largest wart was injected. Injections were administered at 3-week intervals until either complete clearance or a maximum of three treatment sessions. The same wart was injected at all visits in every patient.

### Group B (DPCP group)

In this group, all the patients were subjected to DPCP solution (manufacture date: Feb. 2018, expiry date: Feb. 2021, Batch No. B22018, origin: Merck KGaA) at a concentration of 0.1% applied to the upper inner arm to induce sensitization using a cotton-tipped applicator. The application site was examined the following week for any eczematous reaction. Once the patient was sensitized, DPCP was applied to the mother wart with concentration starting from 0.1% and gradually increased (up to 2%) until a mild eczematous reaction was noticed. Application was performed weekly until the lesion cleared or completion of a maximum of five treatment sessions. The concentration of DPCP was adjusted according to the severity of the inflammatory reaction from the previous application.

### Evaluation

All patients were evaluated at the beginning of the study and at each follow-up visit. The final response was assessed after 4 weeks of the last session for any signs of clearance of the treated and adjacent warts and all complications were recorded. Moreover, two blinded dermatologists were asked to evaluate the response by scoring photos of treated lesions before therapy and 4 weeks following the last session. The observers were asked to score the degree of improvement in number and size of treated and adjacent untreated warts as follows: 0 = no change, 1 = less than 25% improvement, 2 = 25–50% improvement, 3 = 51–75% improvement and 4 = 76–100% improvement. Patient scores of 0 or 1 were considered as unresponsive.

## Statistical analysis

Data were statistically described in terms of mean ± standard deviation (± SD), median and range, or frequencies (number of cases) and percentages when appropriate. Numerical data were tested for the normal assumption using Shapiro–Wilk test. Comparison of numerical variables between the study groups was done using Mann–Whitney *U* test for independent samples. For comparing categorical data, Chi-square (*χ*^2^) test was performed. Exact test was used instead when the expected frequency was less than 5. Correlation between the different variables was done using Spearman equation. Accuracy was represented using the terms sensitivity, specificity, + ve predictive value, –ve predictive value, and overall accuracy. Two-sided *p* values less than 0.05 were considered statistically significant. All statistical calculations were done using computer program IBM SPSS (Statistical Package for the Social Science; IBM Corp, Armonk, NY, USA) release 22 for Microsoft Windows.

## Results

Forty patients completed the study, while 10 patients dropped due to irritability from the sensitization reaction induced by *Candida* antigen and/or DPCP at the start of therapy. There was no significant difference between the two groups regarding participants’ demographic and clinical data including wart type, duration, or recalcitrance (Table 1).

**Table 1:** Demographic, clinical characteristics and treatment outcomes of studied patients

			Candida group *n* = *20*	DPCP group *n* = *20*	*p*- value	
Sex	Female		6 (30.0%)	11 (55.0%)	0.200	
	Male		14 (70.0%)	9 (45.0%)		
Age	Mean ± SD Range		30.00 ± 7.167 19 – 46	30.10 ± 6.480 20 – 42	0.963	
Occupation	None Laborer Office Student Housewife Others		1 (5.0%) 7 (35.0%) 8 (40.0%) 1 (5.0%) 1 (5.0%) 2 (10.0%)	1 (5.0%) 3 (15.0%) 10 (50.0%) 1 (5.0%) 3 (15.0%) 2 (10.0%)	0.740	
Family history	Positive		3 (15.0%)	2 (10.0%)	0.633	
Recalcitrance	Positive		14 (70%)	14 (70%)		1.000
Wart duration (weeks)	Mean ± SD Range		9.925 ± 9.796 1.5–36	11.350 ± 9.826 1–36		0.550
Number of warts	Single Multiple		11 (55.0%) 9 (45.0%)	5 (25.0%) 15 (75.0%)		0.105
Type of warts	Common Filiform Flat Periungual Plantar		9 0 0 2 9	8 2 1 0 9		0.281
Clearance of mother wart	17/40		12 (60%)	5 (25%)		0.54
Clearance of adjacent wart	3/26		3/10 (30%)	0/16 (0%)		0.46
Improvement	None < 25% 25–50% 51–75% > 76%	5 4 9 5 17	2 2 3 2 11	3 2 6 3 6		0.580
Number of sessions	Mean ± SD Range		2.55 ± 0.759 1–3	3.85 ± 1.040 2–5		0.000

*p*-value > 0.05: Non Significant; *p*-value < 0.05: Significant; *p*-value < 0.01: Highly significant

**Fig.1:**
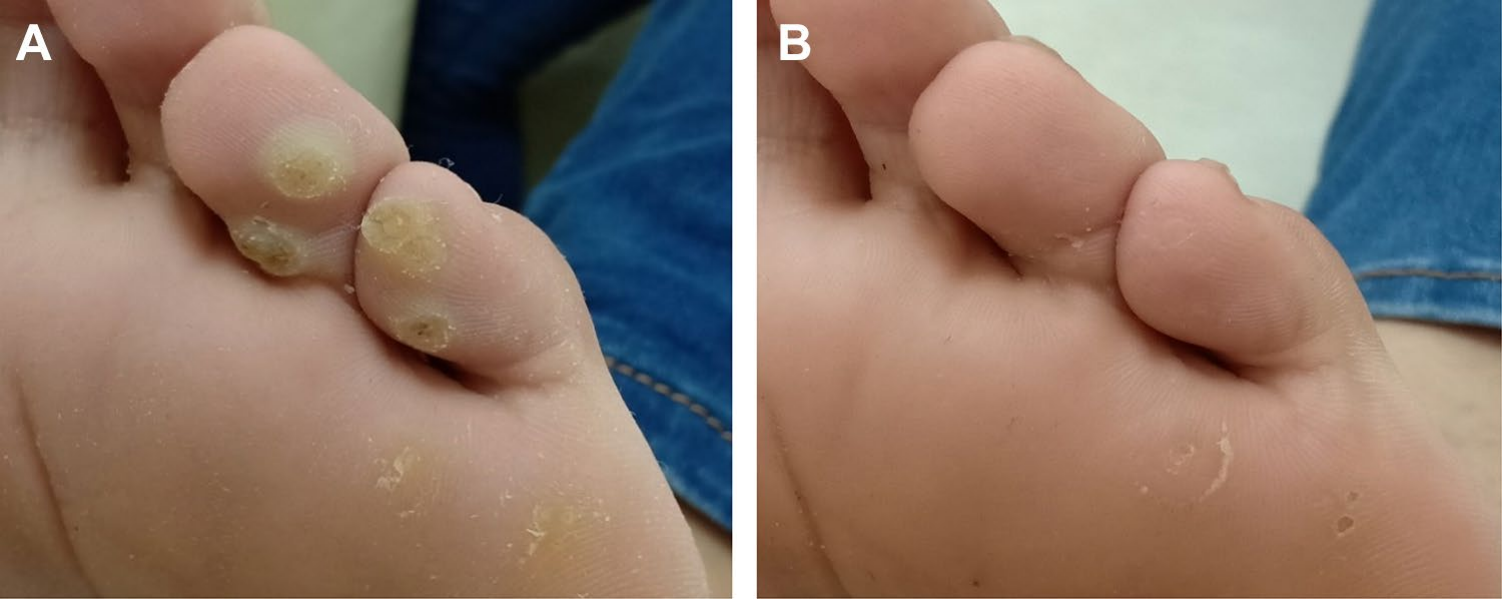
Patient no. 1 before treatment (**A**) and complete clearance of treated warts as well as adjacent warts after two sessions of *Candida* antigen injection (**B**)

**Fig. 2:**
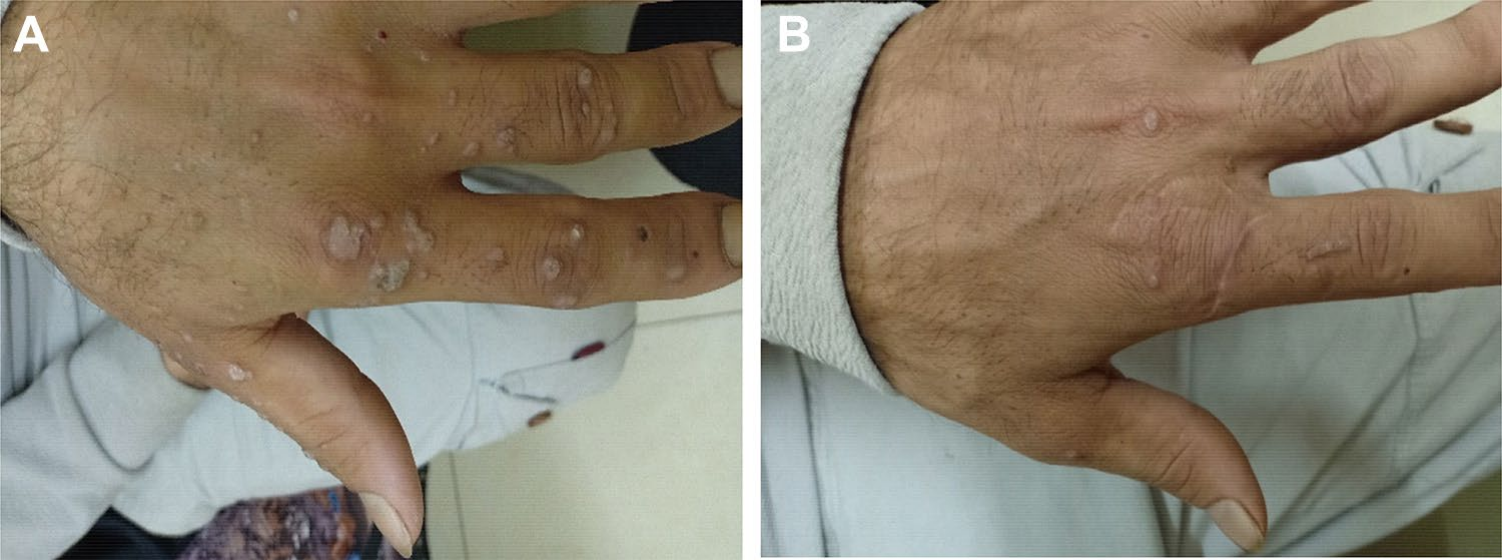
Patient no. 13 before treatment (**A**) and clearance of treated wart as well as several adjacent warts after two sessions of *Candida* antigen injection (**B**)

**Fig. 3:**
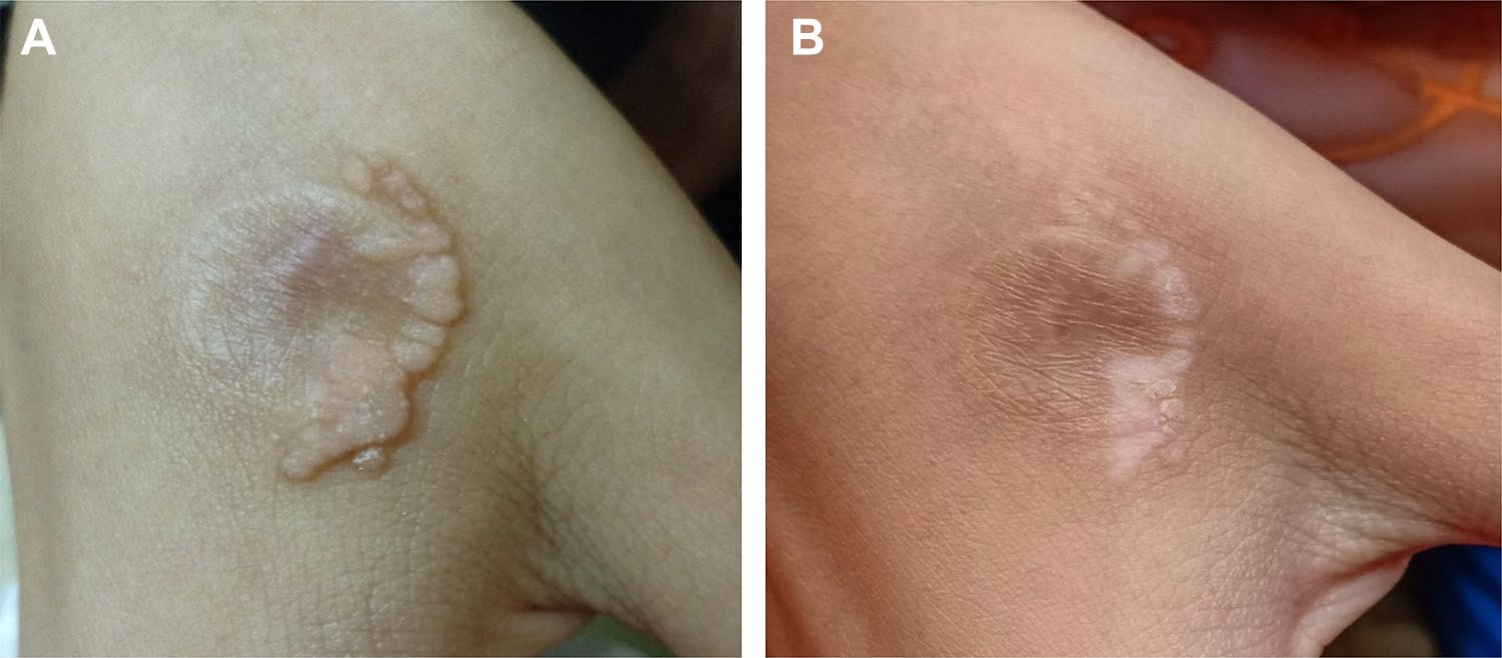
Patient no. 22 before treatment (**A**) and clearance of treated warts after three sessions of DPCP application and also showing DPCP side effect of post-treatment hypopigmentation (**B**)

**Fig.4:**
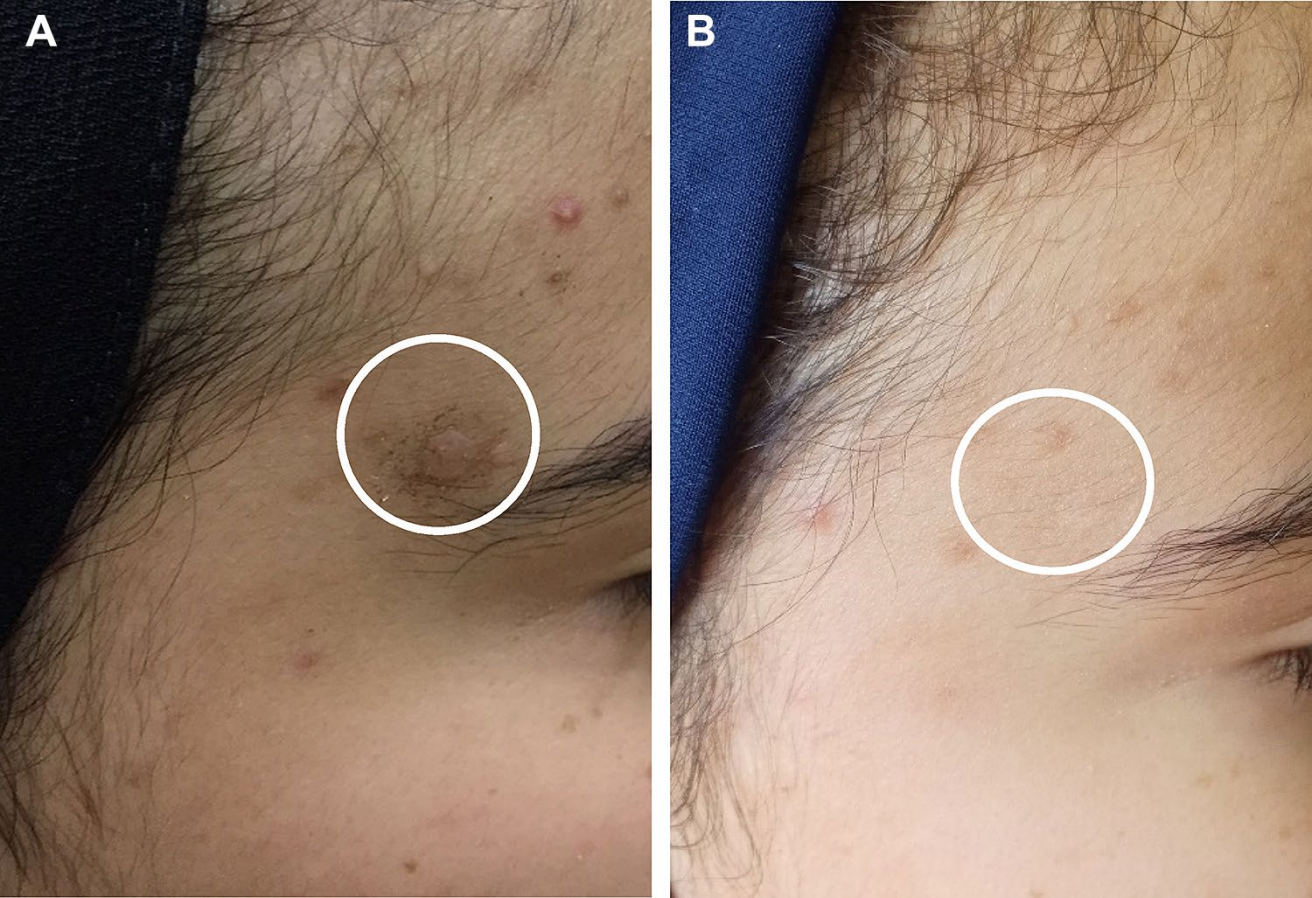
Patient no. 29 before treatment (**A**) and clearance of treated warts after five sessions of DPCP application (**B**)

For all recruits, 17/40 (42.5%) patients showed complete clearance of the central treated wart, with 12/20 (60%) patients in the *Candida*-treated group (Figs. 1, 2, supplementary Figs. 1, 2, 3), and 5/20 (25%) patients in the DPCP-treated group (Figs. 3, 4; supplementary Figs. 4, 5, 6), but this was not statistically significant (95% CI 1.037–5.555, *p* = 0.054). Similarly, there was no significant difference in observers’ evaluation scores for improvement between the *Candida* antigen and DPCP groups (*p* = 0. 580). Twenty-six recruits had warts adjacent to the treated mother wart. Only 3/10 (30%) patients in the *Candida*-treated group and 0/16 patients in the DPCP antigen-treated group showed clearance of their adjacent warts and this was statistically significant (*p* = 0.046) (Table 1).

For all recruits, there was no statistical difference in treatment outcomes as assessed by the median observers’ score for improvement between cases with plantar warts and those with other types of warts (*p* = *0.385*).

### ***Relation between duration and treatment outcomes (Table ******2******)***

**Table 2:** Relation between wart duration and treatment outcomes

All cases		Candida group		DPCP group	
Duration in weeks (range, mean ± SD)	1–36 10.638 ± 9.711	Cleared mother wart, *n* = *12*	No clearance of mother wart, *n* = *8*	Cleared mother wart, *n* = *5*	No clearance of mother wart, *n* = *15*
		1.5–12 4.458 ± 3.215	4–36 18.128 ± 10.776	1–20 6.800 ± 7.596	2–36 12.867 ± 10.232
		*p* = 0.001		*p* = 0.188	
		Cleared adjacent wart, *n* = *3*	No clearance of adjacent wart, *n* = *7*	Cleared adjacent wart, *n* = *0*	No clearance of adjacent wart, *n* = *16*
		1.5–8 3.833 ± 3.617	8–36 19.571 ± 10.517	1–36 13 ± 10.152	
		*P* = 0.022		–	

*p*-value > 0.05: Non Significant; *p*-value < 0.05: Significant; p-value < 0.01: Highly significant

For the *Candida* antigen treatment group, patients with shorter wart duration showed a significantly higher clearance rate of mother and adjacent warts (*p* = 0.001 and 0.022, respectively), while this was not observed in the DPCP contact therapy group. Similarly, non-responders (less than 25% improvement according to observers’ scores for improvement) had significantly longer disease duration than responding patients in the *Candida* antigen-treated group (*p* = 0.021), but not within the DPCP-treated group (*p* = 0.612).

Moreover, a significant negative association between the number of sessions and observers’ score for improvement was detected in the *Candida* antigen treatment group (*r* = – 0.605, *p* = 0.005), but not in the DPCP treatment group (*r* = – 0.090, *p* = 0.707).

Recalcitrant warts showed a significantly longer duration (*p* = 0.000), worse observers’ scores for improvement (*p* = 0.018) and less post-intervention tenderness (*p* = 0.009) in comparison to non-recalcitrant warts. There was no significant difference in improvement scores between recalcitrant and non-recalcitrant warts within neither the *Candida* nor the DPCP-treated groups (*p* = 1.00 and 0.260 respectively). No statistical difference in observers’ improvement scores for recalcitrant warts could be detected between *Candida* antigen and DPCP treatment groups (*p* = 0.678).

### Side effects (supplementary Tables 1,2)

Eight patients (40%) in the *Candida* antigen group suffered side effects of treatment (supplementary Fig. 7) in comparison to 20 patients (100%) in the DPCP group (supplementary Fig. 8) and this difference was statistically significant (*p* = 0.002). Redness and vesicle formation were significantly associated with DPCP treatment in comparison to *Candida* antigen (*p* = 0.013 and 0.004, respectively). A significant positive association between the number of sessions and treatment-induced tenderness and redness was observed in the DPCP group only (*p* = 0.012 and 0.035, respectively). Except for ten patients who refused to continue after sensitization (not included in the analysis), side effects in both groups were expected, tolerable, transient and did not necessitate stoppage of treatment in any of the studied patients.

## Discussion

HPV-associated verrucas still pose a therapeutic dilemma because of the possible associated disfigurement, recurrence, or inefficiency of the treatment options available [8]. In the current study, verruca treatment with *Candida* antigen injection or topical DPCP contact therapy resulted in clearance of the treated lesion in 42% of patients with no permanent adverse effects or scarring. Moreover, treatment with the *Candida* antigen was associated with clearing of untreated adjacent warts in 30% of patients.

Immunotherapy for warts seems to alert the immune system about the presence of an antigen that requires an immune reply with a consequent type IV delayed-type hypersensitivity response along with upregulation of IL-1, IL-12, TNF-α, and IFN-γ. This immune response, if elicited, is not specific to the injected antigen, but may also act against wart tissue [9]. Moreover, contact immunotherapy was shown to be associated with a better response in warts of patients treated with squaric acid dibutyl ester (SADBE), specifically IL-4 and IL-10 [9, 10]. Similarly, IL-10 was also reduced in warts of patients treated successfully with PPD [9]. Whether injectable or contact therapy, immunotherapy for warts may exert its therapeutic response through upregulation of IL-1, IFN -γ, and TNF-α and downregulation of IL-4 and IL-10. [9, 11]

Previous studies suggest that intralesional antigen immunotherapy with *Candida* antigen may induce a nonspecific Th1 inflammatory reaction against HPV, as well as an associated specific Th1 response to papilloma virus capsid protein L1 [12–15]. Macrophage migration inhibitory factor (MIF) was shown to be upregulated among those with warts responding to *Candida* antigen injection [16]. Comparatively, topical immunotherapy with contact sensitizers including DPCP is believed to induce a delayed-type hypersensitivity reaction with the production of several cytokines that may stimulate natural killer cells toward wart tissue [17–20].

Our results show a higher clearance rate and a better observers’ scores for improvement in warts treated with the *Candida* antigen vs DPCP. Indeed, 60% of patients in the *Candida* antigen group showed complete clearance of central treated wart, in contrast to 25% of patients in the DPCP group; however, these differences were statistically insignificant. Similarly, the mean observers’ scores for improvement were better for the *Candida* antigen treatment group, but in a statistically insignificant manner. While 30% of our patients showed resolution of untreated warts with *Candida* antigen, this did not occur with DPCP. A larger number of patients may have showed more significant differences.

Similar to our findings, Khurshid et al. 2009 reported that *Candida* antigen was effective in clearing more than 60% of each injected wart in 67% of patients [21]. Other investigators reported that 56–81% of patients showed clearance of treated warts and 56–100% resolution of untreated warts with *Candida* antigen injections [5, 14, 22]. On the other hand, DPCP was previously shown to successfully clear warts in 66.6–82.9% of patients and in 100% of three children with anogenital warts when applied topically to each wart [23, 24]. Except for Van der Steen et al. 1999, no other investigators reported the effect of treatment with DPCP on untreated warts. The latter authors described a case of verruca plantaris that cleared with DPCP treatment associated with “some involution” in untreated warts. [25]

The demographic characteristics of recruited patients including age did not affect the therapeutic response with *Candida* or DPCP. This was in concordance with previous studies with *Candida* antigen [26]; however, Suh et al. reported that the therapeutic efficacy of DPCP gradually decreased with age [20]. The difference in mean age of recruited patients in the latter study in comparison to ours (14.9 Vs 30.1 years respectively) may account for such variance. It may also be related to varying levels of natural immune responses of different age groups, which may partially explain the low therapeutic efficacy of DPCP we encountered among our patients in comparison to the latter authors.

Based on our results, a significant association was observed between wart duration and the therapeutic response in the *Candida* group. This was in accordance with other studies that revealed that there is a significant inverse relationship between the disease duration and therapeutic response [26, 27].

While our result revealed that there was no significant association between wart duration and therapeutic response with DPCP immunotherapy, conversely another study revealed a significant decrease in success rate as disease duration increased, probably because of its lower capacity to stimulate the immune system in patients with long wart duration [28]. This contradiction may be attributed to different study settings where the aforementioned study examined only periungual warts, which is considered as a relative recalcitrance factor.

Patients with recalcitrant warts showed a significantly lower median observers’ score for improvement in comparison to those with non-recalcitrant lesions. Contrastingly, Garza et al. 2015 reported that in their cohort with 80.4% patients suffering from recalcitrant warts, complete clearance was achieved with *Candida* treatment in 70.9%; however, the study included only pediatric age group with a more robust immune response [29]. Hammad and coworkers also did not find an association between recalcitrance and response to *Candida* antigen injection, and suggested that failure to respond to *Candida* injection treatment may be due to elevated complement C3c and TNF-α in sera of non-responding patients [30]. The fact that recalcitrant warts showed significantly lower post-intervention tenderness than their non-recalcitrant counterparts in our cohort may suggest a frail immune response in these patients.

Our results showed that 40% of the *Candida* antigen group suffered side effects of treatment in comparison to 100% in the DPCP group and this difference was statistically significant (*p* value = 0.002). The side effects of both groups were tolerable, transient, and reversible, yet ten patients dropped out because of these; the findings agreed with previous studies [18, 26, 31–35]. As DPCP side effects tend to be more severe in highly sensitized individuals, [18, 35], it is advisable that patients showing severe local reactions on sensitization be closely monitored for adverse events.

Although, to the best of our knowledge, no comparisons between these two modalities could be retrieved, *Candida* antigen previously showed efficacy over isotretinoin, combined *Candida* and isotretinoin [36], photodynamic therapy [37] and 5 flurouracil [38] in the treatment of warts. Other immunotherapeutic agents showed similar results when compared to *Candida* such as combined bivalent human papillomavirus vaccine, [39] measles, mumps and rubella vaccine [40], purified protein derivative [41], herpes zoster vaccine [42] and tuberculin [43], while bleomycin [38], vitamin D [27, 44], cryo-immunotherapy [45] as well as alternating intralesional PPD and *Candida* [46] were superior to *Candida*.

The discrepancy in results among studies, including ours, for both *Candida* antigen and DPCP in the treatment of warts may be related to several factors. Differences in the preparations used, concentrations of antigen/DPCP, vehicle used, frequency of application, age of patients, site and duration of warts, state of recalcitrance and immune response variations among different ethnicities are among the main factors that can be responsible for such discrepancies.

The proper choice of treatment modality for warts has been discussed before [47, 48]. Our hypothesis is that different immunotherapeutic agents might vary in efficacy according to different patient and wart criteria. Hence, we believe designing comparative studies might help physicians deciding which immunotherapeutic agent to choose. According to our findings, *Candida* antigen can be better tolerated with less adverse events, while DPCP can yield better results in older warts although having more side effects.

Our findings should always be read with some limitations in mind. We were only able to assemble a small sample size, which might affect statistical evaluation and conclusions, and the follow-up period was limited so we still do not have a full perspective of the persistence of the results.

## Conclusion

Intralesional *Candida* antigens and contact DPCP immunotherapy are effective in the treatment of verruca with transient side effects that did not include permanent scarring like other destructive methods. For both modalities, *Candida* antigen is shown to be the superior treatment option for untreated adjacent warts, a better response for warts with shorter duration, fewer numbers of required treatment sessions and lower risk of side effects. Although immunotherapy has always been discussed as a backup plan for recalcitrant warts, early treatment with *Candida* antigen injection may yield good results with early intervention.

## Supplementary Information

Below is the link to the electronic supplementary material.

Supplementary file1 (DOCX 18 KB)

Supplementary file2 (DOCX 1041 KB)

Supplementary file3 (DOCX 2034 KB)

Supplementary file4 (DOCX 165 KB)
