# Supplementary material for: The efficacy and safety of intralesional Candida vaccine versus topical diphencyproprobenone in immunotherapy of verruca vulgaris: A randomized comparative study
Source: Arch Dermatol Res. 2022 Oct 17;315(3):583–91. doi: 10.1007/s00403-022-02402-7 (PMC10020255; doi:10.1007/s00403-022-02402-7)
Supplement: Supplementary file 1 — Supplementary file1 (DOCX 18 KB) [file 403_2022_2402_MOESM1_ESM.docx]

**Table 1: Adverse effects of treatment among all participants**

|  | | **Candida group** | **DPCP group** | **Test value*** | **P- value** |
| --- | --- | --- | --- | --- | --- |
| All side effect | Negative | 12 (60.0%) | 0 (0.0%) | 10.000 | 0.002 |
|  | Positive | 8 (40.0%) | 20 (100.0%) |  |  |
| Myalgia | Negative | 18 (90.0%) | 20 (100.0%) | 2.105 | 0.147 |
|  | Positive | 2 (10.0%) | 0 (0.0%) |  |  |
| Headache | Negative | 19 (95.0%) | 20 (100.0%) | 1.026 | 0.311 |
|  | Positive | 1 (5.0%) | 0 (0.0%) |  |  |
| Hypopigmentation | Negative | 20 (100.0%) | 19 (95.0%) | 1.026 | 0.311 |
|  | Positive | 0 (0.0%) | 1 (5.0%) |  |  |
| Tenderness | Negative | 18 (90.0%) | 17 (85.0%) | 0.229 | 0.633 |
|  | Positive | 2 (10.0%) | 3 (15.0%) |  |  |
| Edema/erythema  /eczema | Negative | 12 (60.0%) | 6 (30.0%) | 0.440 | 0.507 |
|  | Positive | 8 (40.0%) | 14 (70.0%) |  |  |
| Lymphadenopathy | Negative | 20 (100.0%) | 17 (85.0%) | 2.105 | 0.147 |
|  | Positive | 0 (0.0%) | 3 (15.0%) |  |  |
| Redness | No | 12 (60.0%) | 9 (45.0%) | 10.701 | 0.013 |
|  | Mild | 8 (40.0%) | 3 (15.0%) |  |  |
|  | Moderate | 0 (0.0%) | 2 (10.0%) |  |  |
|  | Severe | 0 (0.0%) | 6 (30.0%) |  |  |
| Surface area (cm) | Median (IQR) | 2.5 (1 ‒ 17) | 7 (1 ‒ 25) | -0.757≠ | 0.449 |
|  | Range | 1 – 32 | 0.25 – 80 |  |  |
| Vesicles | Absent | 20 (100.0%) | 13 (65.0%) | 8.485 | 0.004 |
|  | Present | 0 (0.0%) | 7 (35.0%) |  |  |

**Table 2: Relation between wart duration and side effect.**

|  | | **Wart duration (weeks)** | | **Test value** | **P-value** | **Sig.** |
| --- | --- | --- | --- | --- | --- | --- |
|  |  | **Median (IQR)** | **Range** |  |  |  |
| Myalgia | Negative | 6.5 (9) | 1 – 36 | 1.715 | 0.086 | NS |
|  | Positive | 20 (16) | 12 – 28 |  |  |  |
| Headache | Negative | 7 (9) | 1 – 36 | 0.784 | 0.433 | NS |
|  | Positive | 12 (0) | 12 – 12 |  |  |  |
| Tenderness | Negative | 8 (8) | 1 – 28 | 1.562 | 0.118 | NS |
|  | Positive | 2 (1) | 2 – 36 |  |  |  |
| Edema/erythema/eczema | Negative | 8 (8) | 2 – 24 | 0.826 | 0.409 | NS |
|  | Positive | 6 (9) | 1 – 36 |  |  |  |
| Lymphadenopathy | Negative | 7 9) | 1 – 36 | 0.031 | 0.975 | NS |
|  | Positive | 7 (2) | 6 – 8 |  |  |  |
| Redness | No | 8 (6 – 20) | 2 – 36 | 6.607 | 0.086 | NS |
|  | Mild | 3 (2 – 12) | 1 – 16 |  |  |  |
|  | Moderate | 10 (8 – 12) | 8 – 12 |  |  |  |
|  | Severe | 5.5 (3 – 12) | 1 – 20 |  |  |  |
| Vesicles | Absent | 7 (4 – 12) | 1 – 36 | -0.411 | 0.681 | NS |
|  | Present | 8 (3 – 12) | 1 – 20 |  |  |  |
