# Supplementary material for: The efficacy and safety of intralesional Candida vaccine versus topical diphencyproprobenone in immunotherapy of verruca vulgaris: A randomized comparative study
Source: Arch Dermatol Res. 2022 Oct 17;315(3):583–91. doi: 10.1007/s00403-022-02402-7 (PMC10020255; doi:10.1007/s00403-022-02402-7)
Supplement: Supplementary file 2 — Supplementary file2 (DOCX 1041 KB) [file 403_2022_2402_MOESM2_ESM.docx]

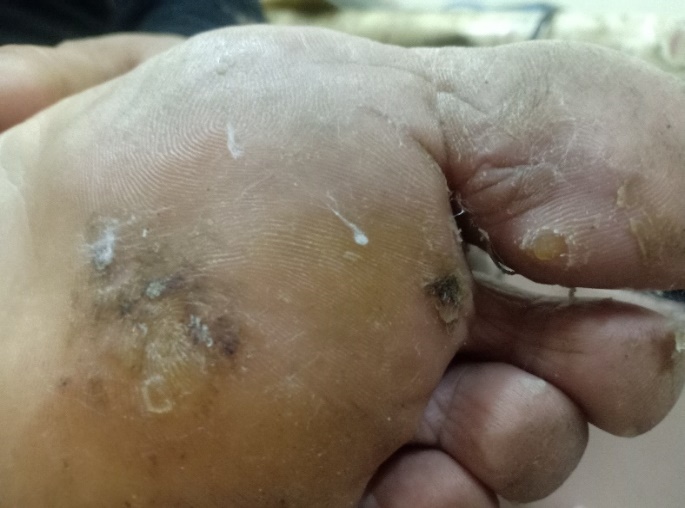

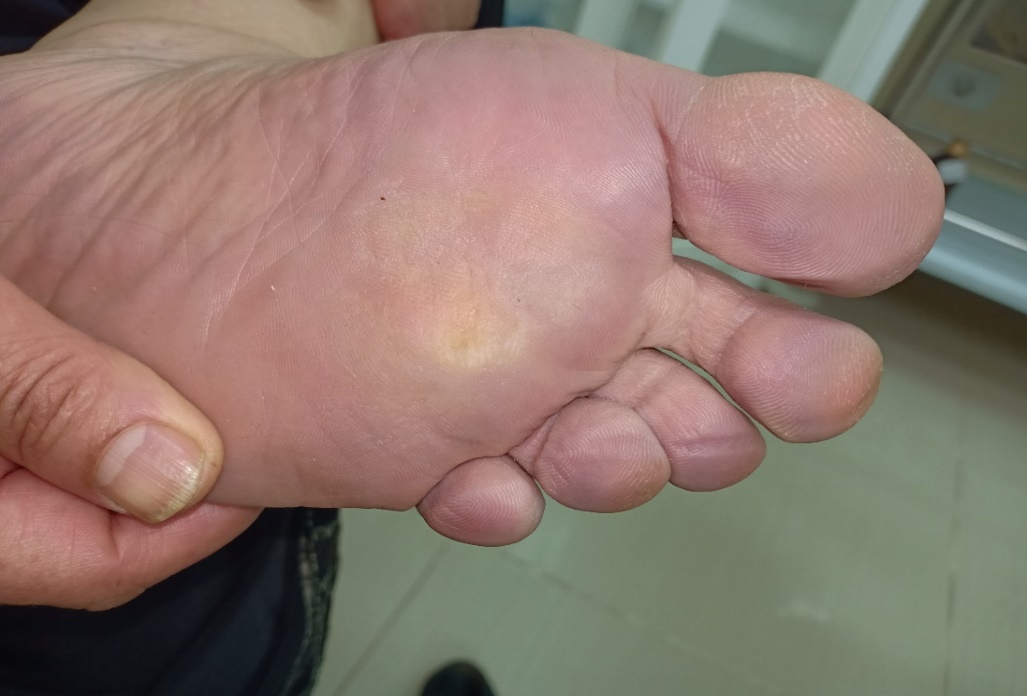


**B**

**A**

**B**

**A**

**Fig.1: Patient no.5 before treatment (A) and clearance of treated and adjacent warts after 3 sessions of candida antigen injection (B)**


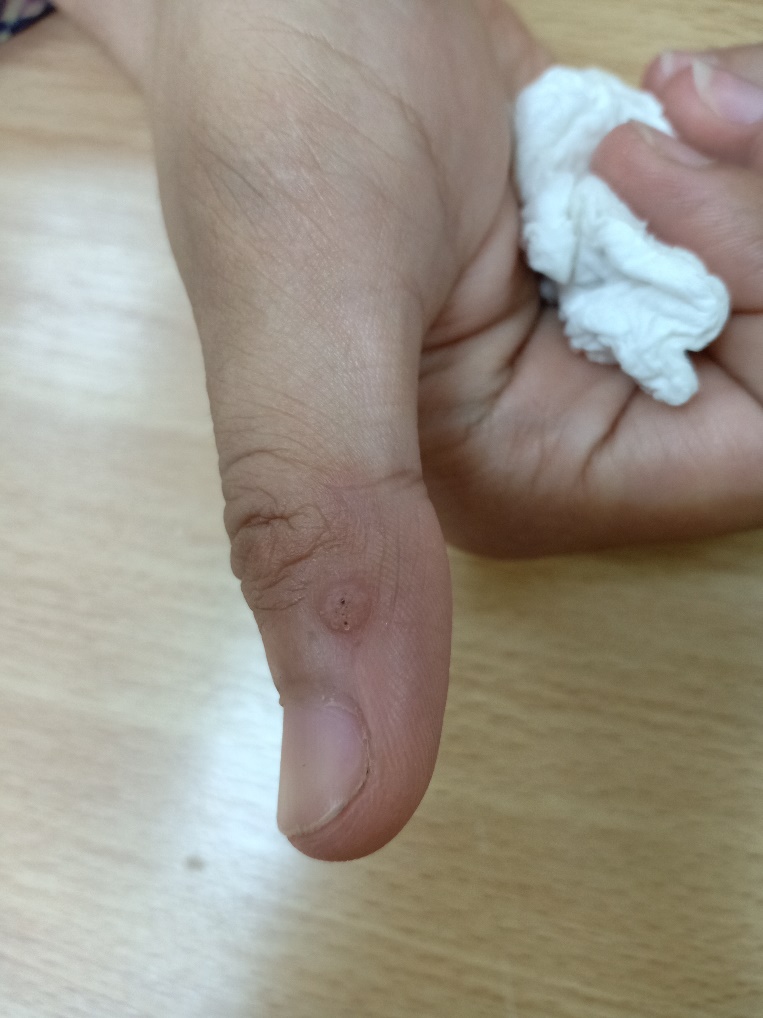

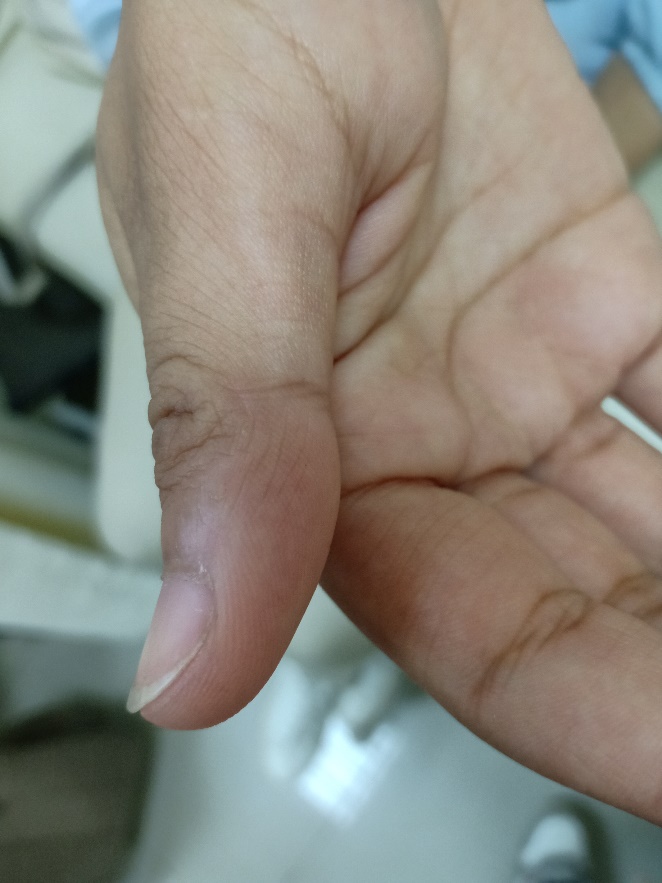


**B**

**A**

**Fig.2: Patient no.8 before treatment (A) and complete clearance of treated wart after one session of candida antigen injection** (B).


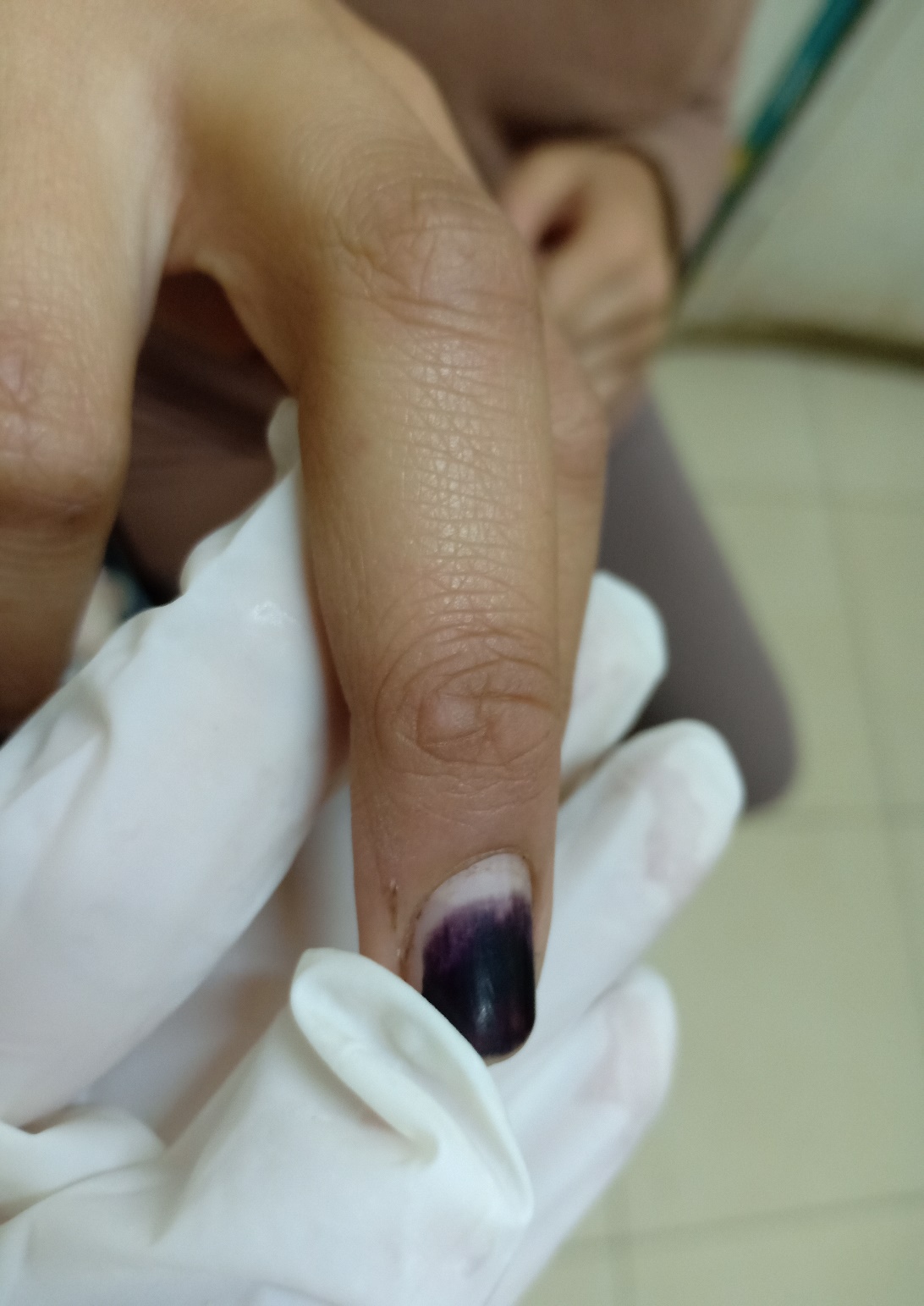

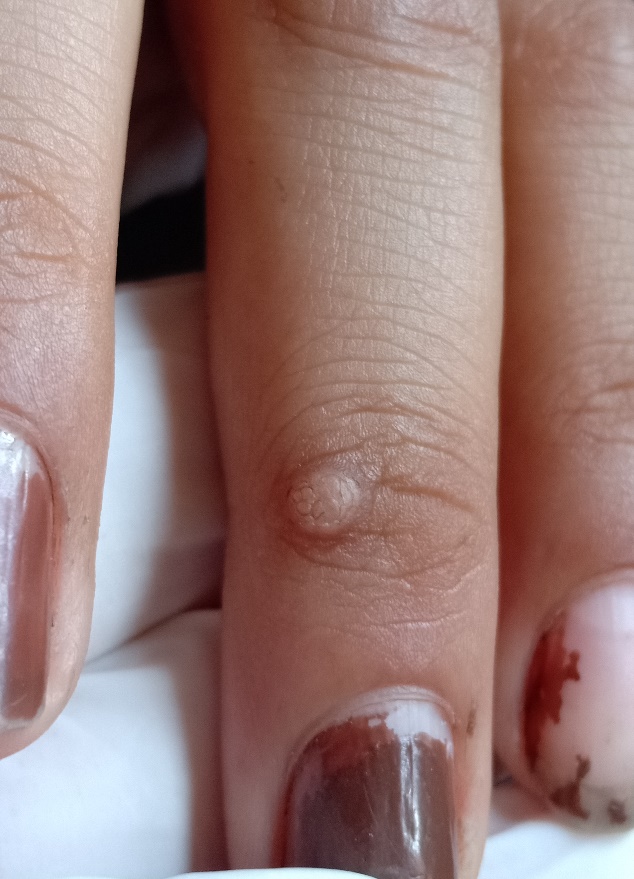


**B**

**A**

**Fig 3: Patient no.14 before treatment (A) and complete clearance of treated wart after 3 sessions of candida antigen injection (B)**
