# Supplementary material for: The efficacy and safety of intralesional Candida vaccine versus topical diphencyproprobenone in immunotherapy of verruca vulgaris: A randomized comparative study
Source: Arch Dermatol Res. 2022 Oct 17;315(3):583–91. doi: 10.1007/s00403-022-02402-7 (PMC10020255; doi:10.1007/s00403-022-02402-7)
Supplement: Supplementary file 3 — Supplementary file3 (DOCX 2034 KB) [file 403_2022_2402_MOESM3_ESM.docx]

**B**

**A**


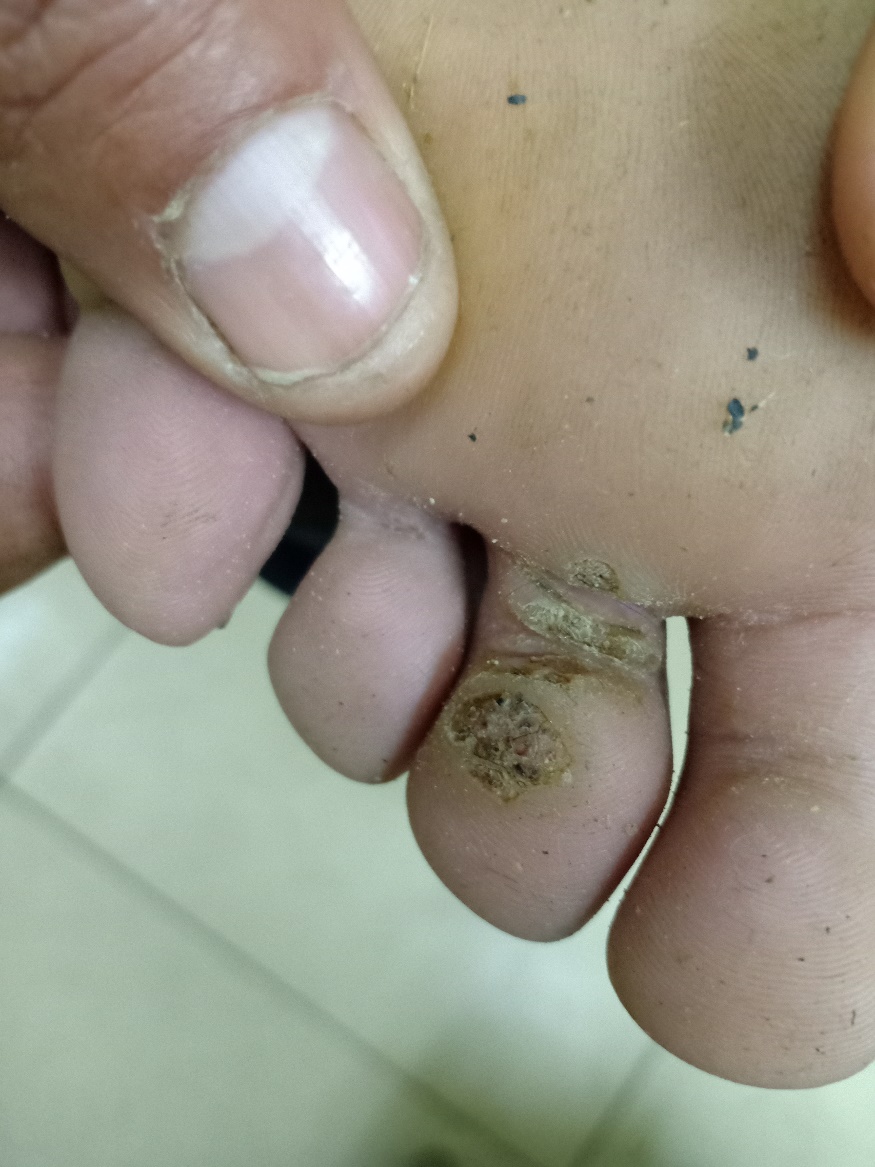

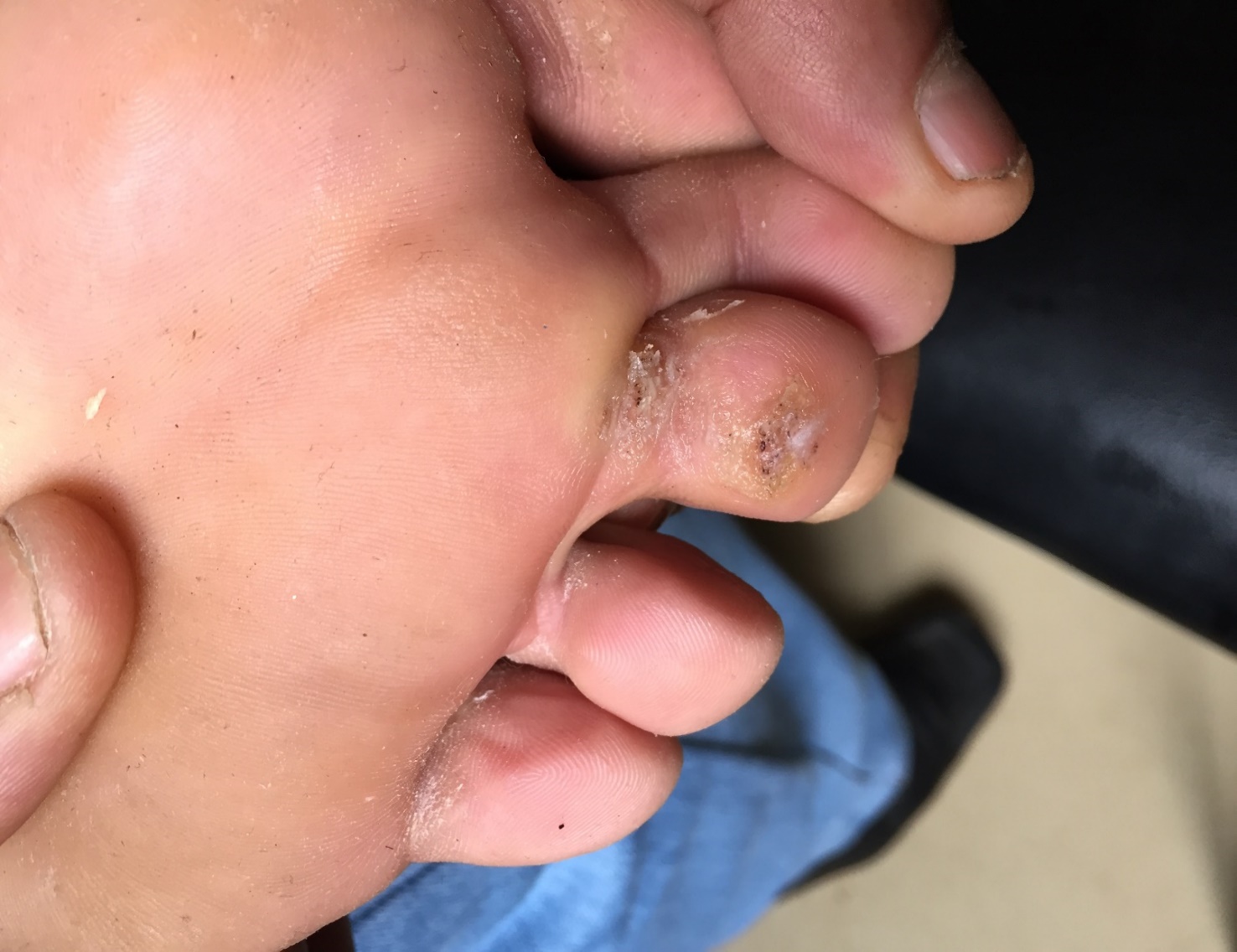


**Fig.4: Patient no.25 before treatment (A) and improvement of treated warts (25% - 50%) after 5 sessions of DPCP application (B)**


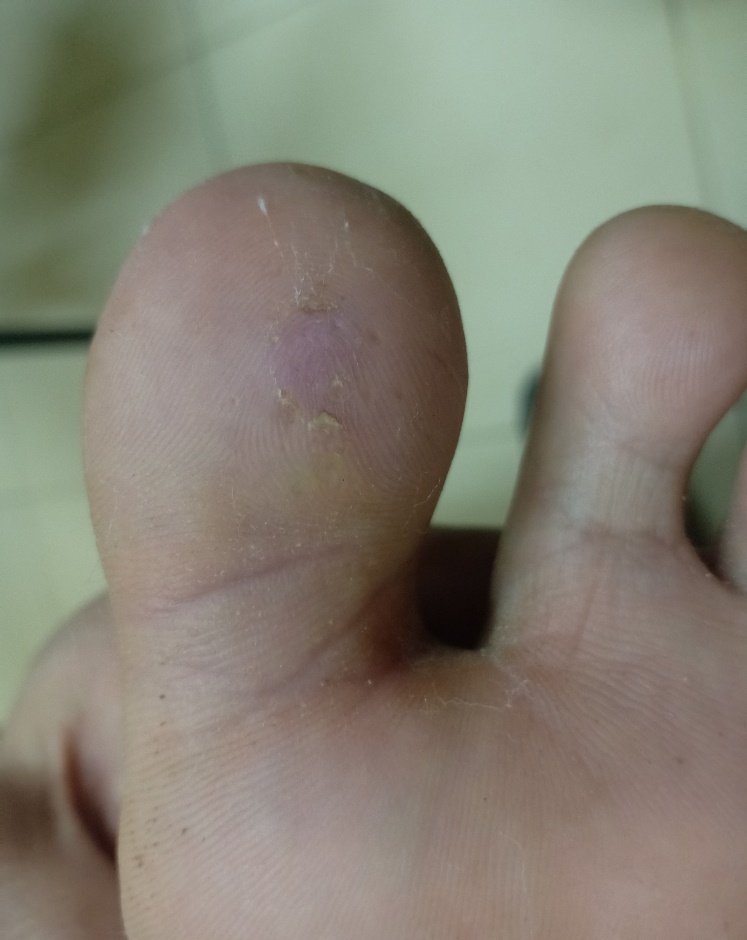


**A**


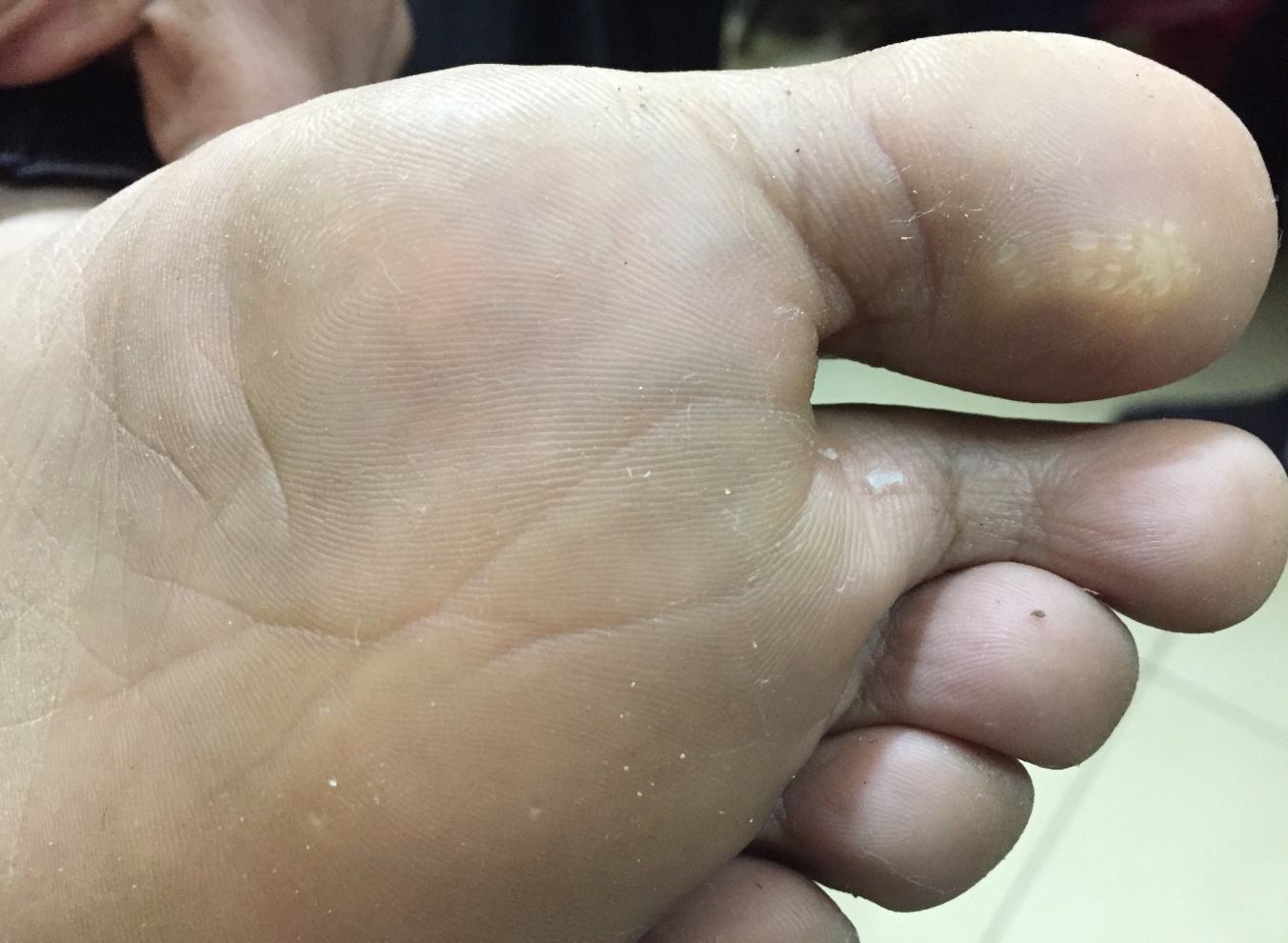


**B**

**Fig.5: Patient no.28 before treatment (A) and clearance of treated warts after 2 sessions of DPCP application** (B)


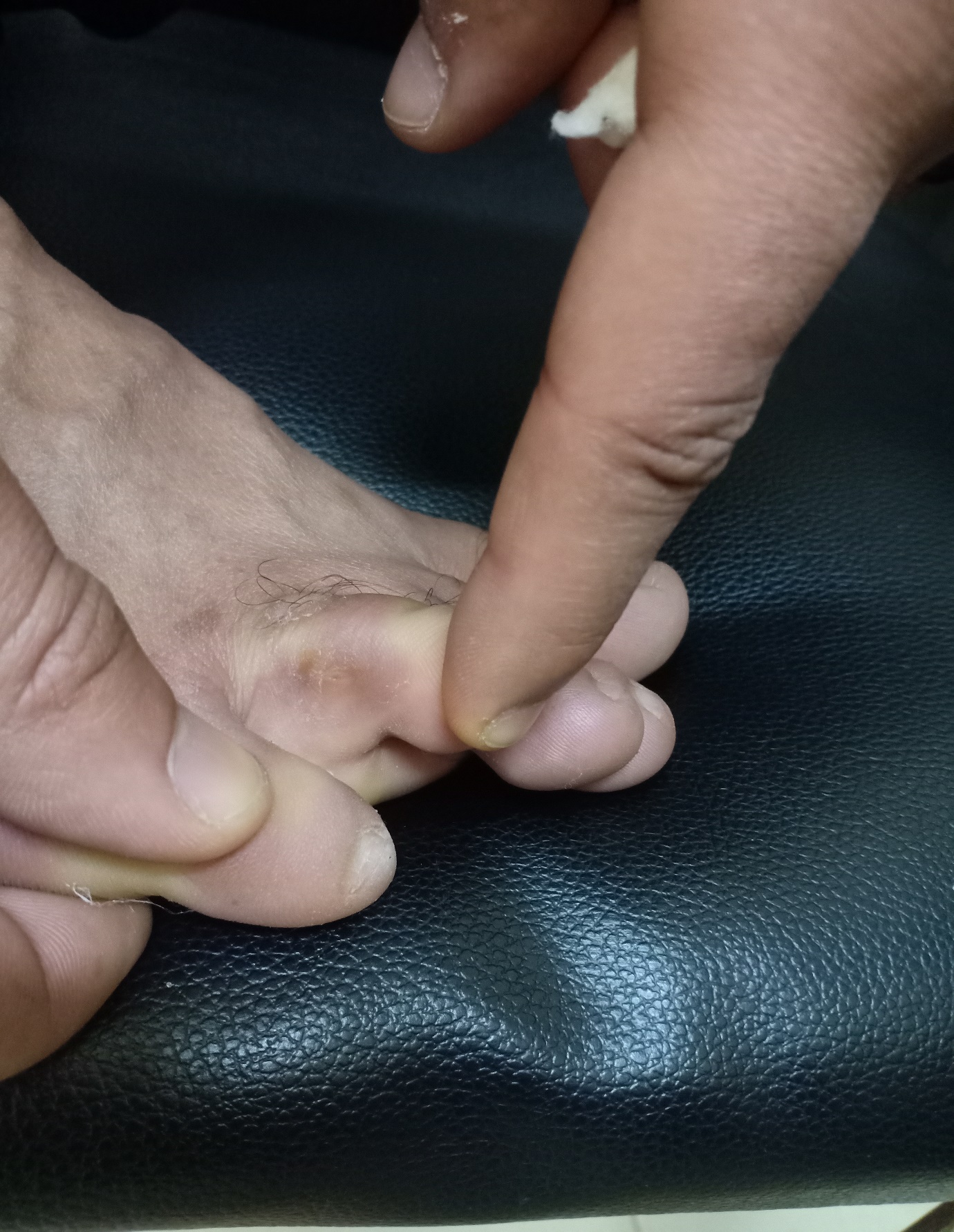

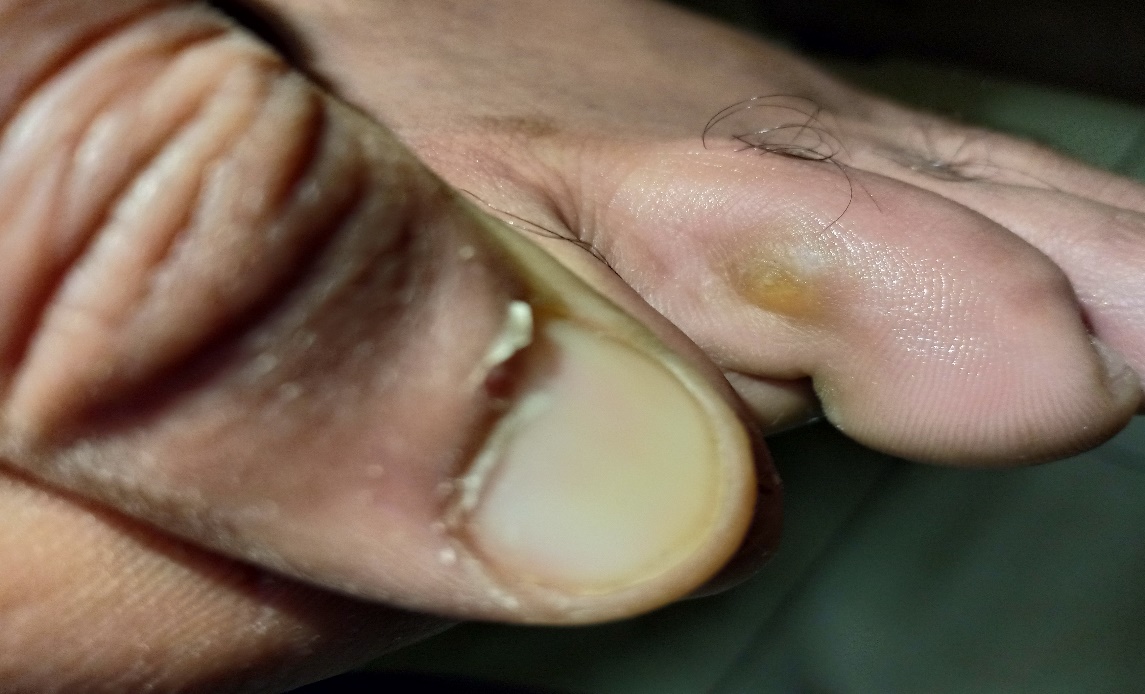


**B**

**A**

**Fig.6: Patient no.33 before treatment (A) and improvement of treated warts (75% - 100%) after 5 sessions of DPCP application (B)**
