# Supplementary material for: The efficacy and safety of intralesional Candida vaccine versus topical diphencyproprobenone in immunotherapy of verruca vulgaris: A randomized comparative study
Source: Arch Dermatol Res. 2022 Oct 17;315(3):583–91. doi: 10.1007/s00403-022-02402-7 (PMC10020255; doi:10.1007/s00403-022-02402-7)
Supplement: Supplementary file 4 — Supplementary file4 (DOCX 165 KB) [file 403_2022_2402_MOESM4_ESM.docx]

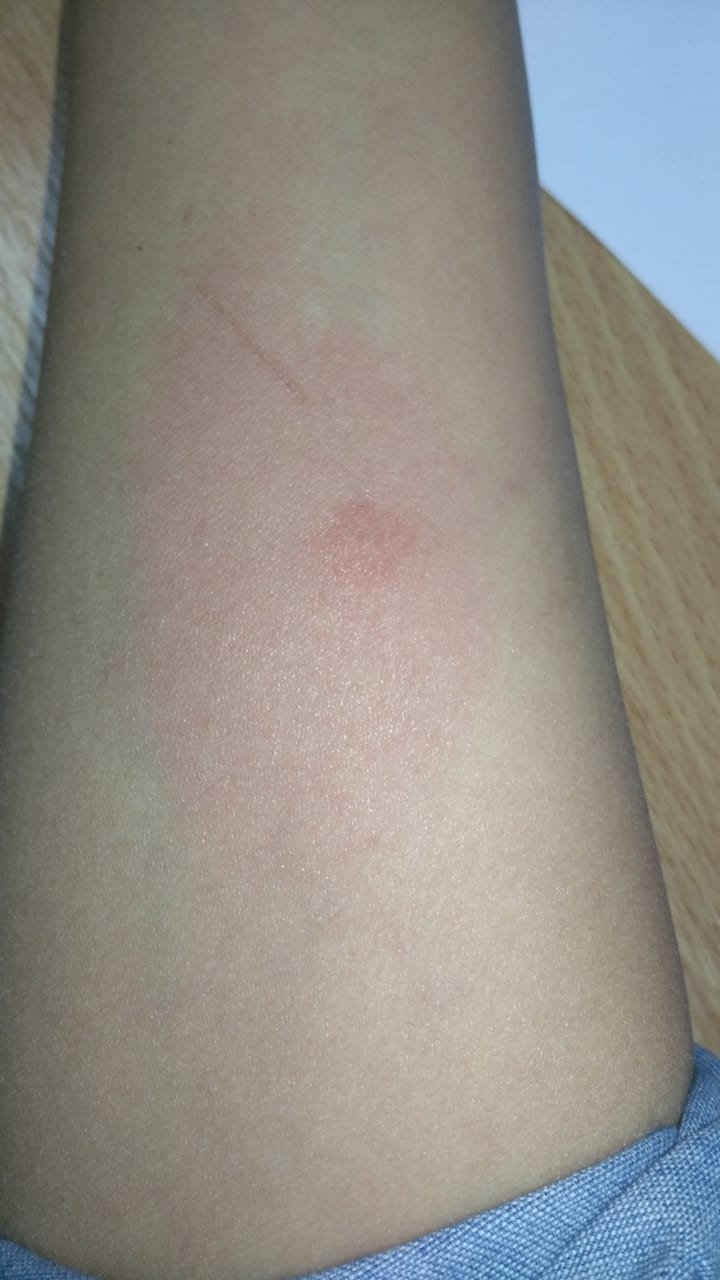
**Fig 7: reaction to candida antigen in patient no.8**


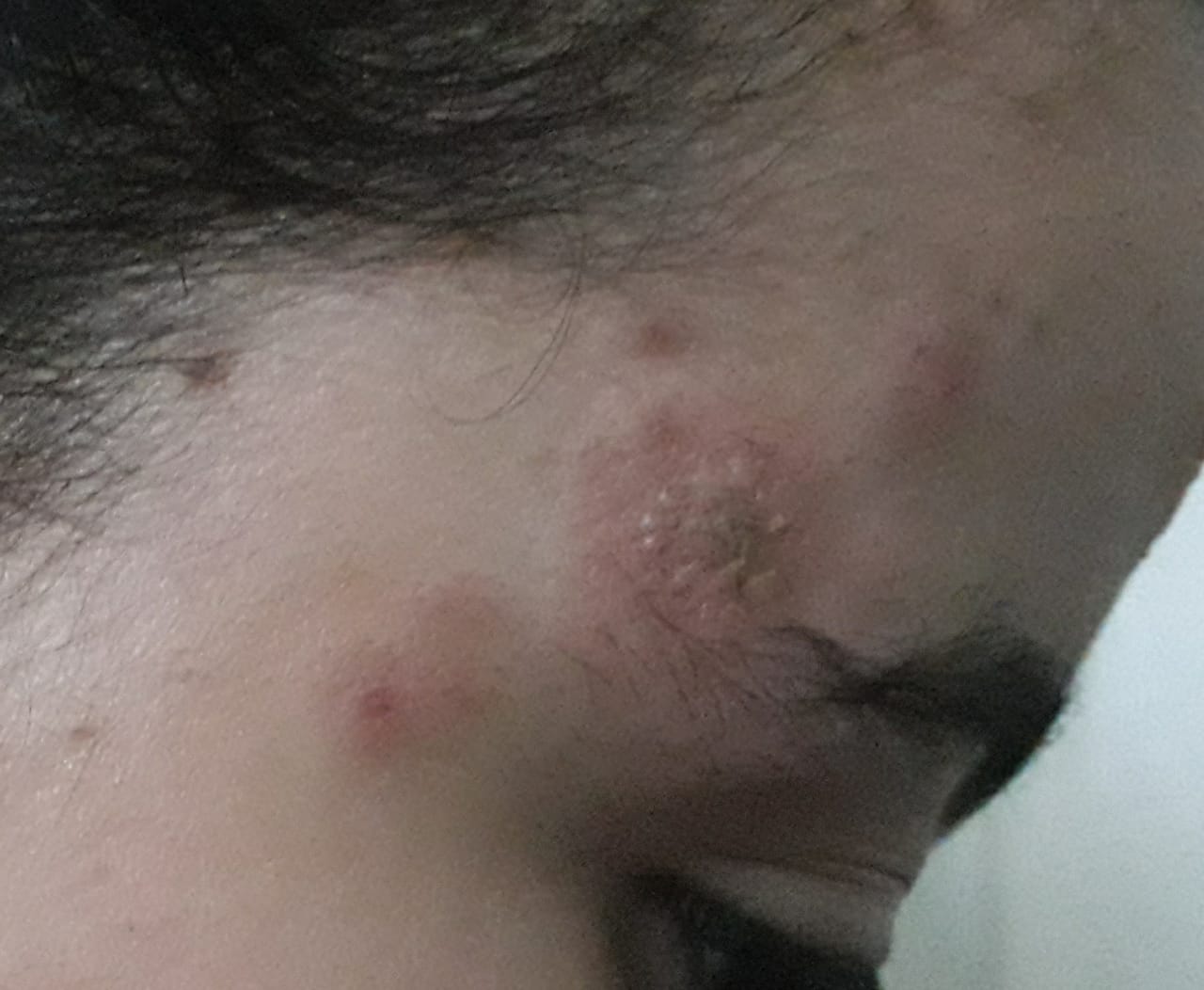


**Fig. 8: reaction to DPCP application in the form severe redness and vesiculation in patient no.29**
